# Vital rates of two small populations of brown bears in Canada and range‐wide relationship between population size and trend

**DOI:** 10.1002/ece3.7301

**Published:** 2021-03-10

**Authors:** Michelle L. McLellan, Bruce N. McLellan, Rahel Sollmann, Heiko U. Wittmer

**Affiliations:** ^1^ School of Biological Sciences Victoria University of Wellington Wellington New Zealand; ^2^ Southwest British Columbia Grizzly Bear Project D'Arcy BC Canada; ^3^ Department of Wildlife, Fish, and Conservation Biology University of California Davis Davis CA USA

**Keywords:** brown bear, carnivore conservation, grizzly bear, population recovery, small population, survival, *Ursus arctos*

## Abstract

Identifying mechanisms of population change is fundamental for conserving small and declining populations and determining effective management strategies. Few studies, however, have measured the demographic components of population change for small populations of mammals (<50 individuals). We estimated vital rates and trends in two adjacent but genetically distinct, threatened brown bear (*Ursus arctos*) populations in British Columbia, Canada, following the cessation of hunting. One population had approximately 45 resident bears but had some genetic and geographic connectivity to neighboring populations, while the other population had <25 individuals and was isolated. We estimated population‐specific vital rates by monitoring survival and reproduction of telemetered female bears and their dependent offspring from 2005 to 2018. In the larger, connected population, independent female survival was 1.00 (95% CI: 0.96–1.00) and the survival of cubs in their first year was 0.85 (95% CI: 0.62–0.95). In the smaller, isolated population, independent female survival was 0.81 (95% CI: 0.64–0.93) and first‐year cub survival was 0.33 (95% CI: 0.11–0.67). Reproductive rates did not differ between populations. The large differences in age‐specific survival estimates resulted in a projected population increase in the larger population (*λ* = 1.09; 95% CI: 1.04–1.13) and population decrease in the smaller population (*λ* = 0.84; 95% CI: 0.72–0.95). Low female survival in the smaller population was the result of both continued human‐caused mortality and an unusually high rate of natural mortality. Low cub survival may have been due to inbreeding and the loss of genetic diversity common in small populations, or to limited resources. In a systematic literature review, we compared our population trend estimates with those reported for other small populations (<300 individuals) of brown bears. Results suggest that once brown bear populations become small and isolated, populations rarely increase and, even with intensive management, recovery remains challenging.

## Graphical abstract

This research investigates population dynamics in two adjacent but genetically and geographically distinct, threatened brown bear (*Ursus arctos*) populations in southwestern British Columbia, Canada, following the caseation of hunting in that area. Differences in independent female and cub survival between the populations resulted in population increase in the larger, partially connected population, and population decrease in the smaller, isolated population. We suggest that when populations are small and isolated, population growth is rare and, even with intensive management, becomes prohibitive for population recovery.

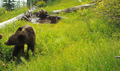

## 1 INTRODUCTION

Many of the world's terrestrial large carnivore populations are declining (Ripple et al., 2014). In most cases, the principal causes of decline are habitat loss, habitat fragmentation, as well as human‐caused mortality from legal harvest, conflict with humans for safety or livestock, and persecution. Independently or synergistically, these factors erode the geographic ranges of species, contracting and fragmenting populations, leaving increased interface edges and population isolates (Henschel et al., 2014; Kenney et al., 2014; Proctor et al., 2012; van Oort et al., 2011). At some point along the continuum of decline, the initial causes may become outweighed in their effect by additional threats arising as a function of diminishing population size. Threats associated with small population sizes include an increased vulnerability to demographic stochasticity, loss of genetic variability, and Allee effects (Berec et al., 2007; Brook et al., 2008; Caughley, 1994).

Successful conservation requires an understanding of the initial causes of decline and those that might be specific to small, remnant populations (Brook et al., 2008; van de Kerk et al., 2019). Many threatened large carnivore species are wide‐ranging, occur at low‐density, and have long generation times. Therefore, even in large populations, it unavoidably takes years to collect sufficient sample sizes of vital rates to infer population trends and their underlying mechanisms (e.g., Gough & Kerley, 2006; Regehr et al., 2018; Schwartz et al., 2006). For small populations, with inevitably small sample sizes, acquiring data required for strong inferences is improbable (Mosnier et al., 2015; Zipkin & Saunders, 2018). As a result, estimates of vital rates and causes for their suppression are seldom obtained in wild populations with few individuals (e.g., Tosoni et al., 2017; Wittmer et al., 2005), despite their world‐wide relevance for the development of effective conservation strategies.

**FIGURE 1:**
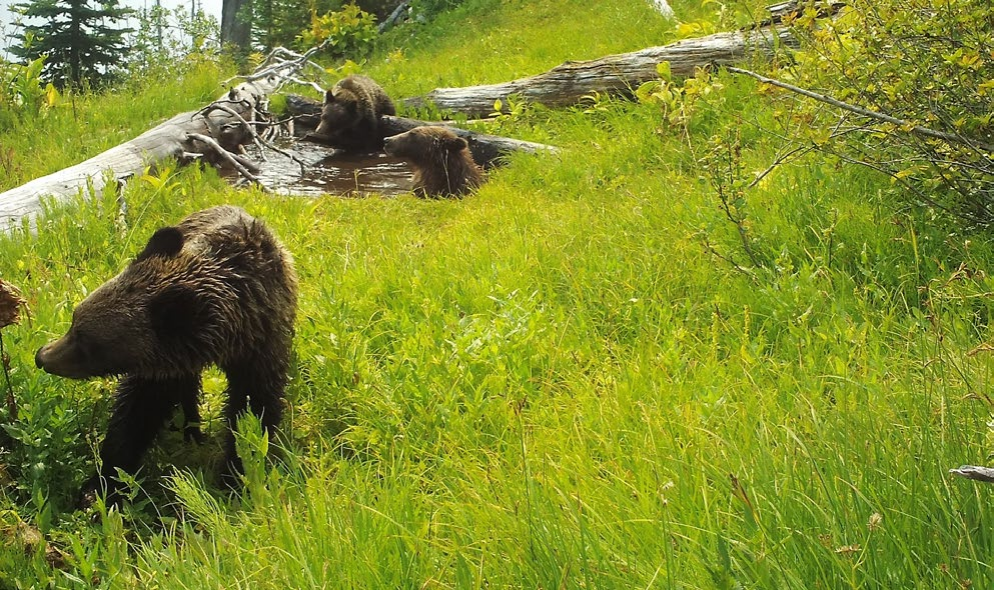
Mother brown bear and offspring in a waterhole. These bears are part of the growing McGillvary Mountains population in southwest British Columbia, Canada

Brown bears (*Ursus arctos;* Figure 1), called grizzly bears over most of North America, are large‐bodied, long‐lived omnivores with late onset of reproduction. Females are predominantly philopatric and therefore do not rapidly colonize neighboring habitats or provide demographic rescue to small populations, while males will often disperse outside of their natal home range (McLellan & Hovey, 2001; Proctor et al. 2004). Brown bears usually reach maturity between 4 and 6 years of age, and females remain with dependent offspring for two or more years (Garshelis et al., 2005; Mace et al., 2012; McLellan, 2015; Schwartz et al., 2006). Brown bears typically have high (>95%) annual adult survival probabilities even as populations approach carrying capacity, and population regulation occurs due to variable recruitment rates that reflect the abundance of food or other density‐dependent effects (Keay et al., 2018; McLellan, 2015; Schwartz et al., 2003; van Manen et al., 2015). As a result, recovery efforts for this species usually target adult female survival, which is often reduced by human‐caused mortality.

Substantial range contraction has resulted in a fragmented global population of brown bears with many small isolates in need of conservation attention (Mattson & Merrill, 2002; McLellan et al., 2017; Zedrosser et al., 2001). There have been several successful efforts to recover such brown bear populations. For example, in the 1930s, as few as 130 brown bears remained in Sweden but following a reduction in human‐caused mortality, the population grew to approximately 700 by the mid‐1990s (Swenson et al., 1995), and to over 3,200 bears by 2008 (Kindberg et al., 2011). In the United States, brown bear populations in both the Yellowstone Ecosystem and in northern Montana grew at up to 7.6% annually for over 20 years (Mace et al., 2012; van Manen et al., 2015) to over 700 bears in each population (Haroldson et al., 2014; Kendall et al., 2009) following the reduction of human‐caused mortality.

Not all recovery attempts of brown bear populations, however, have resulted in marked population increases. In Italy, the relict Apennine bear population has been isolated from other brown bears for more than 1,000 years (Benazzo et al., 2017) and in recent times, consistently numbered between 50 and 60 individuals without evidence of increase notwithstanding persistent conservation efforts (Gervasi et al., 2017). In the French Pyrenees, brown bear populations declined to <10 individuals in 1990, and, despite efforts to reduce adult mortality, by 1995 only five individuals remained in the western part of the range and the central population required reintroduction with bears from Slovenia (Chapron et al., 2003). Reasons for variable outcomes of recovery efforts in brown bears remain poorly understood but may be related to the small size and increasing isolation of remnant populations.

Here, we use data collected from telemetered brown bears to estimate causes of mortality, age‐specific survival probabilities, reproductive rates (age of primiparity, litter size, and interbirth interval), and population growth in two adjacent but genetically separate populations at the southern edge of brown bear distribution along the Coast Mountains of British Columbia, Canada. Previous research using genetic‐based mark–recapture monitoring of these populations from 2005 to 2017 identified divergent population trends following the cessation of legal hunting (i.e., human‐caused mortality) in 2000 (McLellan et al., 2019). Specifically, the larger, higher density McGillvary Mountains population, with some genetic and demographic connectivity to other large populations, was estimated to increase at 2% each year while the adjacent, small (<25 bears) and long‐isolated (Apps et al., 2014; McLellan et al., 2019) North Stein‐Nahatlatch population declined by approximately 5% each year. We then compare our population growth estimates to those published for other small (<300 individuals) brown bear populations across their distribution using a literature review. Combined, our two objectives aim to better understand the relationship between population size, connectivity, and vital rates in brown bears, contributing not only to our understanding of the effectiveness of brown bear recovery efforts but also to the limited empirical knowledge on small population demography in general.

## 2 METHODS AND MATERIALS

### 2.1 Study area

**FIGURE 2:**
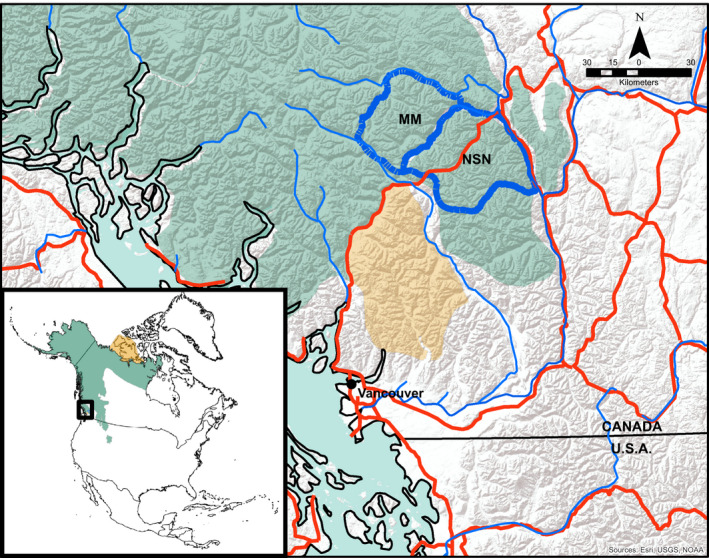
Extant (green) and vagrant (yellow) brown bear distribution in North America (inset) and southwest British Columbia, Canada. The geographic boundary between the genetically distinct McGillvary Mountains (MM) and North Stein‐Nahatlatch (NSN) brown bear populations bisects the study area (blue dash) centered at 50.579181, −122.400917. Highways (red lines), major rivers (blue lines), international border (bottom solid black line)

The McGillvary Mountains and North Stein‐Nahatlatch populations are located in the Coast Mountains of British Columbia, Canada (Figure 2). The McGillvary Mountains population has approximately 45 resident bears, and one‐quarter of its geographic perimeter was connected to other bear populations to the north, while the North Stein‐Nahatlatch population has fewer than 25 individuals and appeared genetically isolated (Apps et al., 2014; McLellan et al., 2019). The western portion of the study area is a temperate rain forest dominated by western red cedar (*Thuja plicata*) and western hemlock (*Tsuga heterophylla*). At high elevations, the winter snowpack is deep and a maritime climate results in moderate summer temperatures. The eastern portion of the study area is warmer and dryer; Douglas‐fir (*Pseudotsuga menziesii*) dominates low elevation forests, and higher elevation forests consist mostly of Engelmann spruce (*Picea engelmannii*) and subalpine fir (*Abies lasiocarpa*) ranging eastward from wet variants of the forest to very dry variants (MacKenzie, 2012). Alpine ecosystems range from lush, moist herbaceous meadows to dry heather (*Cassiope* spp.) and rock dominated topography. The average annual rainfall is approximately 1,030 mm on the western side and 400 mm on the eastern sides of the study area. Avalanche chutes are common throughout the study area and are often used by bears in the spring and early summer (McLellan & McLellan, 2015). Mean road density is 0.21 and 0.16 km/km^2^ in the McGillvary Mountains and North Stein‐Nahatlatch populations, respectively, and each has similar amounts of settled lands along their perimeters.

### 2.2 Bear capture and monitoring

We captured, collared, and monitored bears from 2005 to 2018. Bears were immobilized by darting from a helicopter (see McLellan & McLellan, 2015). Capture was carried out in spring, shortly after den emergence when bears were feeding in avalanche chutes and open alpine meadows, or early autumn when they fed on huckleberries (*Vaccinium membranaceum*). Once immobilized, we weighed, measured, and fitted bears over 2 years old with either GPS or VHF collars (Lotek Inc.). We also obtained a tissue sample for genetic identification and a vestigial premolar for determining age via cementum annuli. We classified 2 to 5‐year‐old female bears as subadults and those 6 years and older as adults (Garshelis et al., 2005). Insufficient data were collected from males to include in this analysis. All collars had canvas spacers to ensure that the collar would drop off, and we weakened the canvas on the collars of subadult bears to ensure they dropped in about 1 year. The Animal Care Committee of the British Columbia Fish and Wildlife Management Branch approved and permitted all capture and handling protocols.

Capture effort, defined as the time we spent searching for bears, was evenly distributed between the study populations until 2014 when it had become apparent that the North Stein‐Nahatlatch population was not only small, but had an unusually high incidence of adult female mortality. Although no bears had been injured or killed in our research, the risk of additional female mortality due to capture was deemed too high, so captures from 2015–2018 were limited to the McGillvary Mountains population. We continued to monitor the vital rates of all collared bears in both populations until 2018.

Throughout the first part of the study (2005–2008), we located collared bears by fixed‐wing aircraft every 2 weeks from May to November. On each flight, we downloaded GPS location data and attempted to visually locate each bear and count the dependent offspring of females. If we did not find a bear for more than 8 consecutive weeks, we censored them from the analyses estimating survival and reproductive parameters at the time of their last known status. The populations were also monitored using genetic capture–recapture, and all females were genetically detected after their collars had dropped or stopped working; therefore, all had known fates while collared. In the second part of the study (2010–2018), some females were fit with VHF collars to reduce the number of recaptures needed to maintain continuity of monitoring. Collared bears were subsequently located and observed from a helicopter at least once each spring and then again in summer and fall. We intermittently located bears from the ground between aerial attempts. Offspring age was determined by size for cubs (bears <1‐year‐old) and yearlings. We grouped attendant offspring that were 2 years of age and older because we could not visually distinguish between 2 and 3‐year‐old bears with certainty if the previous year's status was unknown.

All collars were programmed to signal if the collar had not moved in 24 hr, and these mortality signals were investigated as soon as possible after detection. Whenever we found a dropped collar with rotted canvas, we assumed the bear was still alive. If a bear was found dead, we performed an investigation and necropsy in the field to determine the cause of death.

### 2.3 Survival analysis

We estimated survival (S) for adult and subadult female bears using the staggered entry design for the Kaplan–Meier estimator (Pollock et al., 1989). We considered population (S~Pop) and age class (S~Age) as categorical covariates in candidate models along with the null model (S~1) because we were interested in establishing whether the previously identified differences in population trend (McLellan et al., 2019) were indicative of differences in population‐specific survival and because survival often differs among age classes (Mace et al., 2012; McLellan, 2015; Schwartz et al., 2006). We used months as our monitoring interval from April to October when most bears were active (McLellan & McLellan, 2015) and amalgamated November through March into one monitoring interval because monthly mortality would not be distinguishable when bears were hibernating. We used RMark v.2.25 (Laake & Rexstad, 2008; White, 2008) to fit models and compared models using AIC. Models that deviated from the top model with <2 ∆AIC units were averaged to obtain survival estimates for each population and age class (Burnham & Anderson, 2004). For consistency with analysis of reproductive parameters (below), we also tested for statistical significance of effects present.

We estimated cub survival by observing the number of cubs for each collared female shortly after den emergence and at least once before the following denning season. We assumed cub mortality if they were no longer seen with their mothers. We censored from analysis bears with cubs not located within a month of den emergence or mothers that dropped their collars or that were not visually located again that year. Due to a possible lack of independence of cub survival within a litter (Mace et al., 2012; Swenson et al., 2001), we first tested whether individual cub survival differed from survival within litters using an ANOVA and then tested for a difference in cub survival between populations. We resampled with replacement (bootstrapped) cub survival data 1,000 times to obtain mean survival and 95% confidence intervals (McLellan, 2015). We used the same method to estimate yearling survival. Analyses were conducted using PopTools (Hood, 2011).

### 2.4 Age of primiparity, litter size, and interbirth interval

We located all telemetered females shortly after den emergence in spring to determine whether they had reproduced and to count the number of offspring.

To estimate the average age of primiparity, we used the techniques developed by Garshelis et al. (1998). This method incorporates data from all monitored nulliparous females, including those not monitored to parturition. For each age, the proportion of females in the sample that reproduced are weighted by the proportion of females in the study population available to have a first litter at each age. The resulting estimates are not biased toward early maturing bears, which, due to shed collars and mortality, are less likely to be lost from the sample than late‐maturing bears. We used the same weighting technique of Garshelis et al. (1998) as adapted by McLellan (2015) to estimate the average interbirth interval for each population. We used data from all collared females for which one or more birthing events were known. The proportion of the female sample that had a subsequent litter during each year following the birth year was used to estimate breeding intervals without bias toward shorter intervals. For each parameter, the means and confidence intervals were obtained for each population by bootstrapping the original sample 1,000 times using PopTools (Hood, 2011).

### 2.5 Reproductive state transition and reproductive rate

The stable reproductive state distribution describes the proportion of adult females in each reproductive state (Schwartz & White, 2008). For brown bears, female reproductive state is defined by the presence and age of dependent offspring: alone (A), with cubs (C), with yearlings (Y), or with 2‐year‐old or older offspring (T). To obtain an estimate of each population's stable reproductive state distribution, we first estimated the probability that a female will transition from one reproductive state to another using the multi‐state model in RMark (Laake & Rexstad, 2008). We considered ten biologically possible transitions that are observable when an adult female is monitored for two or more consecutive years. Because survival and recapture are a prerequisite for observing a transition, their probabilities are set to 1.0. The resulting estimates are used to populate a transition matrix (right in Equation 1) that, when multiplied by a hypothetical starting state matrix (left in Equation 1; in this case all females are alone), gives the reproductive state distribution after one transition (*t2*; Equation 1).

(1) $$t2=\left[\begin{array}{cccc} 1 & 0 & 0 & 0 \end{array}\right]\left[\begin{array}{cccc} \text{AA} & \text{AC} & 0 & 0\\ \text{CA} & \text{CC} & \text{CY} & 0\\ \text{YA} & \text{YC} & 0 & \text{YT}\\ \text{TA} & \text{TC} & 0 & 0 \end{array}\right]$$

We then iteratively multiplied the resulting age distribution by the transition probability matrix in a Markov chain until it reached the asymptotic stable reproductive state distribution. For this analysis, we used markovchain package v. 0.6.9.15 (Spedicato et al., 2014) in program R (v.3.6.1; R Core Team, 2019). We resampled the data with replacement and bootstrapped estimates of the stable reproductive state distribution 1,000 times to estimate means and 95% CIs for each population. We did not have a sufficient sample size to include female age as a covariate for transition probabilities.

We estimated the mean reproductive rate (*m_x_*_;_ female cubs/year/female) for each population by multiplying the estimated number of female cubs per litter by the stable reproductive state proportion of females with cubs (Schwartz & White, 2008). Because the sex of each cub was not determined, we assumed the number of female cubs to be half the total number of cubs. Like all methods that pool data across time, this method for estimating the reproductive rate assumes constant transition probabilities, but is more robust to possible sampling bias from capture and variability in monitoring duration than by simply using the proportion of monitored individuals (Schwartz & White, 2008).

### 2.6 Population growth and stable age distribution

We estimated finite population growth rate (*λ*) by constructing an age‐structured matrix population (Leslie matrix) model using repeated random samplings (Monte Carlo estimation) from the bootstrapped probability distributions of age‐specific survival and reproduction for each population (Mace et al., 2012). We considered the age of last reproduction as 24 years (McLellan, 2015; Schwartz et al., 2003). For each iteration, we solved for the dominant eigenvalue, which is the population growth rate. For each population, we also estimated the net reproductive rate (R_0_), defined as the estimated number of female cubs an adult female will produce in her lifetime, and the mean generation time (GenT), defined as the time required for a typical female to produce R_0_ offspring (Caswell, 2001). Analyses were conducted using the popbio v.2.2.4 package (Stubben & Milligan, 2007) in program R (R Core Team, 2019). We repeated this process 1,000 times for each population to estimate the mean and variance for each latent variable.

We estimated the stable age distribution of each population by converting the age‐structured matrix population model into a stage‐structured population model. Because vital rates were estimated for age groups, only transition rates required calculation. The transition rate is the expected proportion of individuals transitioning from one life stage to the next and is conditional on individual survival and the population growth rate (Caswell, 2001; Fujiwara & Diaz‐Lopez, 2017). We applied a conditional age group‐transition rate described by Caswell (2001) where the probability that an individual will transition from one age group to the next (*P_j,i_*) is:

(2) $$P_{j,i}=\frac{\lambda^{‐\left(x_{j}‐x_{i}‐1\right)}l\left(x_{j}‐1\right)}{\sum_{x=x_{i}}^{x_{j}‐1}\lambda^{‐\left(x‐x_{i}\right)}l\left(x\right)}$$

and *λ* is the population‐specific growth rate, *x_i_* is the first age in stage *i*, *x_j_* is the first age in stage *j* = *I* + 1, and *l* (*x*) is the survivorship at time *x*. We repeated this process for all 1,000 bootstrapped Leslie matrices from the preceding analysis to estimate the confidence interval around each population's stable age class outcome (Mace et al., 2012).

### 2.7 Population growth estimates in small populations of brown bears

To investigate the relationships between population size and population growth rates in small populations of brown bears, we used the IUCN Red List of Threatened Species, which lists all isolated populations globally (McLellan et al., 2017). We limited our search to unhunted populations that had fewer than 300 bears. Then, we used Google Scholar and Web of Science to search for additional information. Specifically, we searched the name of the country or region, the species, and the term “population” (e.g., “Himalaya” + “*Ursus arctos*” + “population”) for each population we had identified above and only included populations for which there was a population size or trend estimate available. For populations with long histories of research and sometimes changing population size, we used the size estimated toward the beginning of population change and did not consider examples from populations that did not have temporally overlapping estimates of size and growth rate. We categorized the populations according to their known geographic or genetic connectivity (connected to other populations or isolated) and history of augmentation (augmented or not).

## 3 RESULTS

Between 2005 and 2018, we captured and collared 25 independent female brown bears in the McGillvary Mountains (*n* = 16) and North Stein‐Nahatlatch (*n* = 9) populations resulting in survival and reproductive data for 43.1 and 26.0 bear‐years (cumulative number of years sampled for all bears) in each population, respectively.

### 3.1 Causes of mortality

No collared females died in the McGillvary Mountains population while 5 collared, independent female bears died in the North Stein‐Nahatlatch population resulting in 0.19 mortalities/bear‐year of monitoring. Causes of mortality for females varied: Two nulliparous females (aged seven and eight, respectively) were illegally killed by humans. Three female bears died of natural causes amounting to 0.12 natural mortalities/bear‐year; one adult (age 18) with an unknown reproductive state was killed in the early spring by another bear; one subadult (age 4) died in her den and was severely emaciated with severe gelatinous bone marrow transformation indicating starvation (Raglus et al., 2019); and one bear (age 20) died late in the fall with no sign of trauma and was suspected to have died due to natural senescence with a maxillary deforming dental abscess possibly a contributing factor.

### 3.2 Survival of independent females

Comparison of known‐fate survival models indicated that survival differed between populations (Table 1), but not age class. Model averaged independent female survival was 1.00 (95% CI: 0.96–1.00) in the McGillvary Mountains and 0.81 (95% CI: 0.64–0.93) in the North Stein‐Nahatlatch population (Table 2).

**TABLE 1:** Selection results of models estimating known‐fate survival probabilities of collared female brown bears between 2005 and 2018 in southwest British Columbia, Canada

Model	*n* ^a^	AICc	Δ AICc^b^	*ω* ^c^
S (~Pop)	2	51.52	0.00	0.978
S (~1)	1	59.71	8.19	0.016
S (~Age)	2	61.70	4.19	0.006

Note

In addition to equal survival among groups (~1), models considered population (~Pop), either the McGillvary Mountains or North Stein‐Nahatlatch population, and age class (~Age), subadults from 2 to 5 years and adults ≥6 years, as factors contributing to survival.

^a^ Number of model parameters.

^b^ Difference between AICc of model and the AICc of the highest ranked model.

^c^ Model weight.

**TABLE 2:** Summary of survival rates by age class estimated using the Kaplan–Meier estimator from monitoring collared brown bears in the McGillvary Mountains (MM) and North Stein‐Nahatlatch (NSN) populations from 2005 to 2018

	MM	NSN	*p* value
Survival			
Cubs	0.85 (0.62–0.95)	0.33 (0.11–0.67)	.004
Yearling	1.00	1.00	
Independent female	1.00 (0.96–1.00)	0.81 (0.64–0.93)	.002
Reproduction			
Litter size	2.33 (1.89–2.78)	2.25 (2.00–2.75)	.837
Proportion females with cubs	0.20 (0.13–0.30)	0.17 (0.07–0.26)	.625
Interbirth interval (years)	4.08 (3.63–4.4.67)	4.50 (4.00–5.00)	0.780
m_6−24_	0.23 (0.16–0.38)	0.19 (0.09–0.31)	

Note

Bootstrapped survival estimates for cubs (first year of life), yearlings (age = 1). Independent females included both subadults (2–5 years) and adults (6–24 years). Reproductive rates for adult females ages 6–24 years (m_6–24_) estimated based on monitoring collared females with cubs. There were no yearling mortalities and therefore no variance estimates.

### 3.3 Age of primiparity, interbirth interval, and litter size

Six of the monitored females in the McGillvary Mountains were nulliparous during the course of the study; three did not reproduce by 6, 7, and 8 years old when they were censored because they had lost their collars, and one female reproduced for the first time at age 11. The remaining two had first surviving litters at age eight and nine. Although both these females were monitored from ages 3 to 7 and 6 to 8, neither was observed the year immediately before having surviving cubs so could have had nonsurviving cubs at ages seven and eight, respectively. Two more females were first captured at age 7 with cubs. The estimated mean age of primiparity was 8.3 years (95% CI: 7.0–10.0) excluding possible nonsurviving first litters. Including the possible nonsurviving first litters, the mean age of primiparity would be 7.9 years (95% CI: 6.1–10.0). In the North Stein‐Nahatlatch, the age of first surviving litter was observed for one bear at 12 years, and two nulliparous females died at ages 7 and 8 years old. We were unable to obtain estimates of primiparity with sufficient precision to compare populations.

Interbirth intervals did not measurably differ between populations. We observed six interbirth intervals and six partial intervals for eight females in the McGillvary Mountains, and four interbirth intervals for three females in the North Stein‐Nahatlatch. The resulting estimates of interbirth interval were 4.08 years (95% CI: 3.63–4.67) and 4.50 years (95% CI: 4.00–5.00), respectively (Table 2).

Litter sizes were also similar in the McGillvary Mountains and North Stein‐Nahatlatch. We estimated litter sizes based on 21 cubs in nine litters ($\bar{x}$ = 2.33, 95% CI: 1.89–2.78) in the McGillvary Mountains and nine cubs in four litters ($\bar{x}$ = 2.25, 95% CI: 2.00–2.75) in the North Stein‐Nahatlatch (Table 2).

### 3.4 Survival of dependent offspring

We estimated the survival rates of cubs and yearlings as well as the reproductive rates of adult females from the reproductive events of 22 adult females (15 McGillvary Mountains, seven North Stein‐Nahatlatch) that produced 20 cubs in eight litters in the McGillvary Mountains (the mother of the remaining litter lost her collar before the fall and thus did not contribute to offspring survival estimates) and nine cubs in four litters in the North Stein‐Nahatlatch. Cub survival was 0.85 (95% CI: 0.62–0.95) in the McGillvary Mountains and 0.33 (95% CI: 0.11–0.67) in the North Stein‐Nahatlatch. Cub survival was independent of litter membership (*p* = .97); in the McGillvary Mountains, three cubs were lost from two litters (2 of 3 and 1 of 2) while all cubs in six other litters survived. In the North Stein‐Nahatlatch, one litter of three was entirely lost, and each of three other litters lost one of two cubs (Table 2). No cub mortalities were attributed to maternal mortality in either population. We monitored the fate of 26 yearlings (20 McGillvary Mountains, six North Stein‐Nahatlatch) and observed no mortalities and therefore were unable to obtain variance estimates for yearling survival.

### 3.5 Stable reproductive state distribution

**FIGURE 3:**
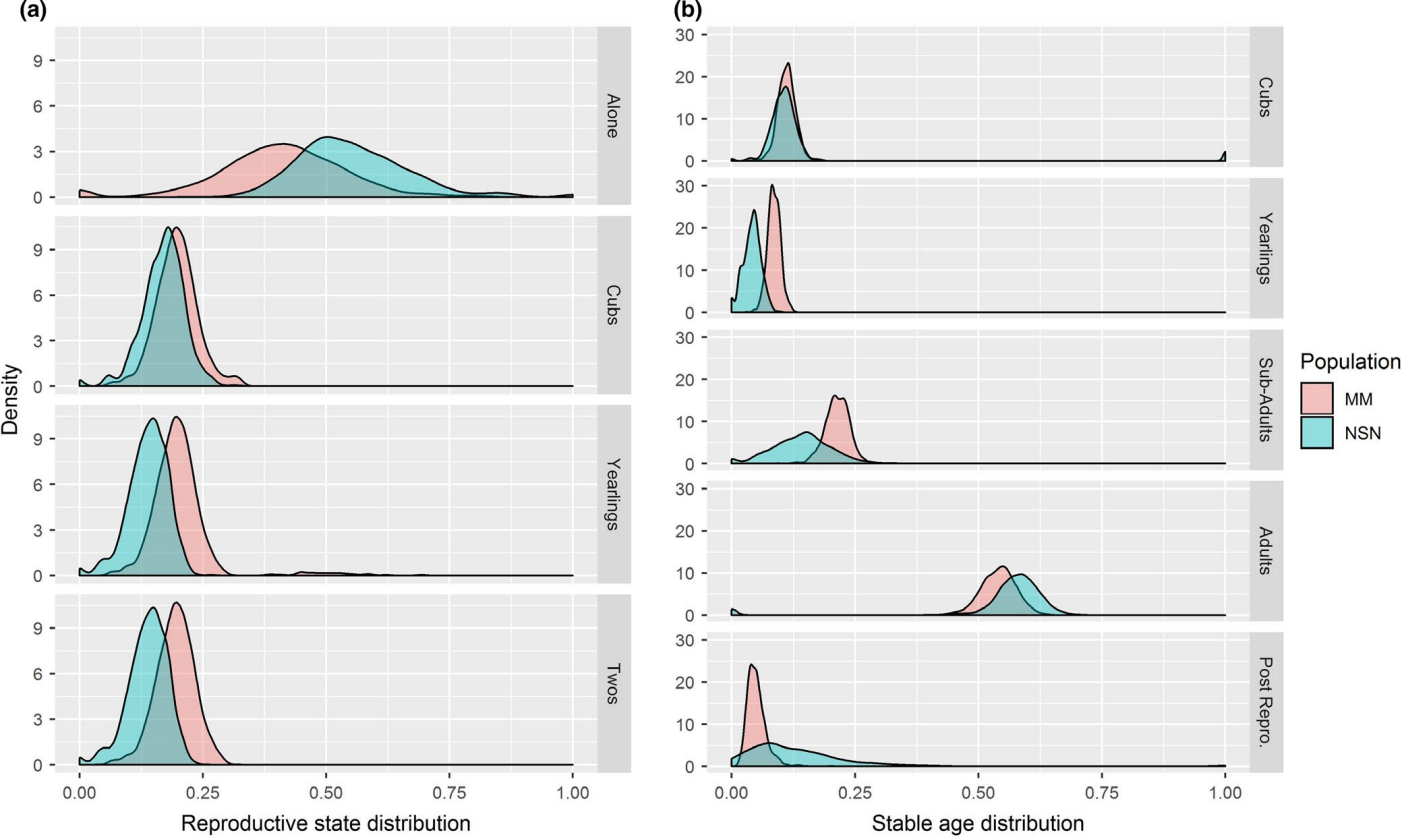
Density plots of (a) the stable reproductive state distribution estimated using in multi‐state transition models on reproductive data from collared adult female brown bears (≥6 years). (b) Bootstrapped estimates of the stable age distribution estimated from vital rates for grizzly bears in the McGillvary Mountains and North Stein‐Nahatlatch populations in southwestern British Columbia, Canada

We observed 38 reproductive state transitions in the McGillvary Mountains (13 bears) and 30 in the North Stein‐Nahatlatch (six bears). The resulting nonparametric, bootstrapped estimates for the stable reproductive state distribution predicted similar proportions of females with cubs in each population, 0.20 (95% CI: 0.13 –0.30) in the McGillvary Mountains and 0.17 (95% CI: 0.07–0.26) in the North Stein‐Nahatlatch, and resulted in mean reproductive rates of 0.23 (0.16–0.38) and 0.19 (0.09–0.31), respectively. Transition probabilities suggested that the proportion of adult females being alone was 0.41 (95% CI: 0.00–0.65) in the McGillvary Mountains and 0.55 (95% CI: 0.38–0.84) in the North Stein‐Nahatlatch (Table 3; Figure 3a).

**TABLE 3:** Reproductive state transition rates (±95% CI) estimated using multi‐state models on reproductive data from collared adult female brown bears (≥6 years) in the McGillvary Mountains and North Stein‐Nahatlatch populations in southwestern British Columbia, Canada

Population	From state	Transfer to state			
		Alone	Cubs	Yearling	Twos
MM	Alone	0.63 (0.38–0.82)	0.38 (0.18–0.62)		
	Cubs	0.00	0.00	1.00	
	Yearling	0.00	0.00		1.00
	Twos	0.75 (0.28–0.97)	0.25 (0.03–0.76)		
NSN	Alone	0.67 (0.41–0.85)	0.33 (0.15–0.59)		
	Cubs	0.17 (0.02–0.63)	0.00	0.83 (0.15)	
	Yearling	0.00	0.00		1.00
	Twos	1.00	0.00		

### 3.6 Stable age distribution

The difference in survival probabilities resulted in differences in the stable age distribution of each population (Figure 3b). The McGillvary Mountains had a higher proportion of yearling and subadult bears, whereas the North Stein‐Nahatlatch had proportionately more adults and older bears (Figure 3b).

### 3.7 Population growth

Mean estimates of projected population growth indicated that, given the estimated stable reproductive state and demographic rates, the McGillvary Mountains population should have grown with *λ* = 1.09 (95% CI: 1.04–1.13) while the North Stein‐Nahatlatch population should have declined with *λ* = 0.84 (95% CI: 0.72–0.95) throughout the study. The mean estimated generation time was 13.2 years (95% CI: 12.6–13.8) in the McGillvary Mountains and 11.7 years (95% CI: 9.2–14.6) in the North Stein‐Nahatlatch. In contrast, the net reproductive rate (R_0_) was over seventeen times higher in the McGillvary Mountains (3.03; 95% CI: 1.66–4.65), than in North Stein‐Nahatlatch (0.17; 95% CI: 0.02–0.53) population.

### 3.8 Population growth estimates in small populations of brown bears

**FIGURE 4:**
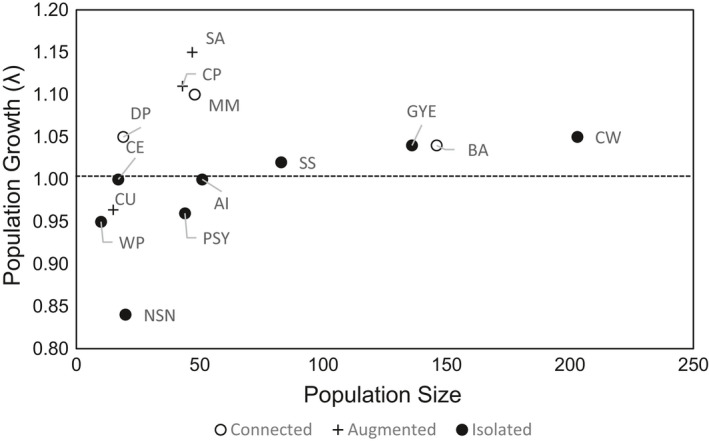
The relationship between brown bear population growth rate (*λ*) and population size from this research and other unhunted populations including isolated populations (●), population with some connectivity to neighboring populations (○), and augmented populations (+). Populations are identified by letters: AI: Apennines, Italy (Gervasi et al., 2017); SA: Southern Alps, Europe (Tosi et al., 2015); BA: Banff, Canada (Garshelis et al., 2005); CU: Cabinet Mountains, USA (Kasworm et al., 2014; Kendall et al., 2016; Proctor et al., 2012; Wakkinen & Kasworm, 2004); CE: Cantabrian East, Spain (Clevenger et al., 1987; Palomero et al., 2007; Pérez et al., 2014); CW: Cantabrian West, Spain (Clevenger et al., 1987; Palomero et al., 2007; Pérez et al., 2014); CP: Central Pyrenees, Spain‐France (Chapron et al., 2009); DP: Deosai, Pakistan (Nawaz et al., 2008); MM: McGillvary Mountains, Canada; NSN: North Stein‐Nahatlatch (This research, McLellan et al., 2019); PSY: Purcell‐South Yaak (Proctor et al., 2012); WP: West Pyrenees, Spain‐France (Chapron et al., 2009); GYE: Yellowstone, USA (Schwartz et al., 2006; van Manen et al., 2015)

Comparing population growth rates with those of other brown bear populations with available data showed that the projected increase in the McGillvary Mountains population was similar to other connected populations following reductions in human‐caused mortality (Figure 4). In contrast, the projected decline in the North Stein‐Nahatlatch population was very low but mirrored observed declines in other small and isolated brown bear populations.

## 4 DISCUSSION

We used data from telemetered individuals in two adjacent but distinct brown bear populations in British Columbia, Canada, to measure the demographic components of population change following the elimination of human‐caused mortality from legal hunting. Although both populations were in ecologically similar environments, one had triple the density and some genetic and demographic connectivity to neighboring bear populations, while the other was small (<25 individuals) and genetically isolated. Despite similar management efforts, the larger, connected population we studied had higher independent female and cub survival rates than the small, isolated population resulting in widely divergent population trends.

### 4.1 Small population inferences

An interesting caveat of studying small populations with very few adult females is that vital rate estimates, and subsequent inferences, were unavoidably drawn from small sample sizes. Extreme outcomes (high and low) are common with small samples, and therefore, researchers have little confidence with small samples obtained from large populations. However, because the number of individuals we monitored approached the size of the entire population, we are confident that our population‐specific estimates and projections are accurate for the period of study, but less confident that the results necessarily reflect systemic factors in the observed trends. Chance events can play a major role in the dynamics of small populations (Engen & Sæther, 1998). If, by chance, a different group of females with the same genetic makeup had been living in the study area, we would likely have generated different estimates. Although we were sampling from an infinite potential population, our sample size was severely restricted to the few animals that were actually there.

### 4.2 Population growth estimates in small populations of brown bears

Comparing data from the two populations we studied with other small, unhunted brown bear populations suggested some relationships among population size, connectivity, and population growth for the species. Although a statistically rigorous comparison was not possible due to the limited availability of vital rate estimates and their variances, small, isolated bear populations below approximately 50 individuals usually continued to decline even with efforts to reduce mortality unless they were augmented (Figure 4). This agrees with observations from other studies of small populations of carnivores. Small populations (*n* = 31 with data) of African wild dogs (*Lycaon pictus*) monitored over 15 years showed that 50% of populations with ~50 or fewer individuals declined to extirpation, while 75% of populations with ~100 individuals increased over the same period (Woodroffe & Sillero‐Zubiri, 2020). The more cryptic Asiatic cheetah (*Acinonyx jubatus venaticus*) has also experienced population declines and likely extirpations of small isolates (Farhadinia et al., 2017).

### 4.3 Survival estimates in small populations of brown bears

Survival rates in the larger, and connected McGillvary Mountains population, were typical of unhunted brown bear populations below carrying capacity. Under such conditions, adult female survival is usually ≥0.95, subadult female survival ranges between 0.81 and 0.96, yearling survival between 0.70 and 0.93, and cub survival between 0.69 and 0.88 (e.g., Garshelis et al., 2005; Mace et al., 2012; McLellan, 2015; Schwartz et al., 2006; Wakkinen & Kasworm, 2004). Adult female survival in the small and isolated North Stein‐Nahatlatch population was much lower (0.81). Other small and isolated brown bear populations also report lower adult female survival rates (range between 0.91 and 0.93) (Chapron et al., 2009; Kasworm et al., 2007; Tosoni et al., 2017). In these populations, despite considerable conservation efforts, female survival was similar or even lower than in several heavily hunted brown bear populations (McLellan, 2015; Miller et al., 1997). High adult female mortality is further illustrated by the high natural mortality rate of 0.12 mortalities/bear‐year of monitoring in the North Stein‐Nahatlatch population; much higher than recorded in large populations where it ranged from <0.005 mortalities/bear‐year monitoring in the Yellowstone Ecosystem (Schwartz et al., 2006) to 0.018 mortalities/bear‐year monitoring in the Canadian Flathead (McLellan, 2015). The low adult female survival in the Stein‐Nahatlatch population is likely hindering population recovery as population viability in long‐lived, slow reproducing species is often heavily affected by adult female survival (de Silva & Leimgruber, 2019; Heppell et al., 2000).

Cub survival in the North Stein‐Nahatlatch population was among the lowest documented for the species. Only a few high‐density, unhunted Alaskan populations in remote areas where they were thought to be at carrying capacity had similarly low cub survival (Keay et al., 2018; Sellers et al., 1999). We were unable to determine the specific causes of cub mortality because only their mothers were monitored directly. However, in addition to stochasticity, three different mechanisms may explain the low cub survival we observed in the North Stein‐Nahatlatch population. First, low cub survival could indicate that the North Stein‐Nahatlatch population is at a low‐density carrying capacity and, at least in some years, limited by food. Second, concurrent long‐term genetic monitoring of our study populations (McLellan et al., 2019) including an associated pedigree analysis identified multiple instances of parent‐offspring and full sibling mating in the North Stein‐Nahatlatch population (unpublished data). Studies measuring the effect of parent‐offspring or full sibling mating found a 33% and 77% reduction in juvenile survival of various mammal species in zoos (Ralls et al., 1988) and wild ungulate populations (Walling et al., 2011), respectively. Third, infanticide by adult males may also have contributed to low cub survival (McLellan, 1994). Sexually selected infanticide has been documented in brown bears in Sweden (Swenson et al., 2001), and it may be exacerbated when the adult sex ratio favors males (Chapron et al., 2009; McLellan, 2005). In small populations with few adult females and long interbirth intervals, there will often be years when all adult females are with dependent offspring, and none are reproductively available (e.g., Gonzalez et al., 2016) likely resulting in an increased chance of infanticide and creating a component Allee effect on juvenile survival as the population decreases.

### 4.4 Reproductive rates in small populations of brown bears

Reproductive rates were similar in both of our study populations but generally lower than in other brown bear populations below carrying capacity (Mace et al., 2012; McLellan, 2015; Zedrosser et al., 2011). The reproductive state distributions of the two populations we studied suggest that adult females in the North Stein‐Nahatlatch population may be without dependent offspring more frequently than in other brown bear populations (e.g., Garshelis et al., 2005; Schwartz et al., 2003; Støen et al., 2006), including the McGillvary Mountains. Long interbirth intervals and late age of primiparity contributed to an increased number of adult females without dependent offspring (offspring ≤2 years) and the associated reduction in reproductive rates. Although the sample sizes used to estimate reproductive parameters in these populations are insufficient to support any definitive conclusions, the observed ages of primiparity in the North Stein‐Nahatlatch were older than the 5–7 years of age at which females in most other populations reproduce (Mace et al., 2012; McLellan, 2015; Schwartz et al., 2006; but see Garshelis et al., 2005).

### 4.5 Small population dynamics

The increased vulnerability of small populations to decline has been widely discussed, concluding that small populations of sexually reproducing diploids are subject to loss of genetic diversity and some form of Allee effect (Berec et al., 2007; Caughley, 1994; Frankham et al., 2019). Further, the effects of demographic stochasticity on population growth and the probability of extinction are increased in small populations. The random fluctuations in birth and death events have very little effect on population growth in large populations; however, in small populations, simultaneous “bad luck” among few individuals can cause the population to decline to zero (Engen & Sæther, 1998). Fortunately, inbreeding and loss of genetic diversity are potentially reversible by restoring gene flow via augmentation or natural immigration (Åkesson et al., 2016; Poirier et al., 2019; Quinn et al., 2019; van de Kerk et al., 2019; Yumnam et al., 2014). An example is the genetic restoration of the Florida panthers (*Puma concolor coryi*) from population augmentation (Johnson et al., 2010) that increased survival of first generation admixed panthers in all age classes (van de Kerk et al., 2019). Natural immigration also appears to be contributing to the recent increase in the Western Cantabrian brown bear population (Pérez et al., 2014). Re‐establishing connectivity with the growing McGillvary Mountains population by conserving unoccupied suitable habitat to geographically connect populations should be a primary focus of conservation management aimed at restoring the North Stein‐Nahatlatch population.

Notwithstanding the prevalence of theory on small population dynamics, there are very few published examples of vital rates from wild populations with fewer than 30 individuals. Our research shows that the survival rates for both juveniles and adults in a small and isolated brown bear population were at the extreme range for what has been observed for the species in larger populations and significantly different from those observed in the neighboring but connected population. Our findings support the theory that as populations decline, we can expect wide variations in vital rates that diverge from those common for the species, potentially exacerbating the rate of decline (Lande, 1998). Also, that small, isolated populations of large carnivores may not respond as well to recovery actions that were successful for larger, or more connected populations of the same species (Fanshawe et al., 1991; Ferreras et al., 2001; Groom et al., 2014; Tosoni et al., 2017). For many carnivores, this means that solely focusing on reducing adult mortality is likely to be insufficient to promote recovery and should not be the only conservation strategy.

By understanding the demographic components of population change, and how they may differ in small populations compared to larger ones, we not only increase our understanding of the relationships between population size and the potential for recovery but also are better able to convincingly prescribe targeted population‐specific recovery initiatives.

## CONFLICT OF INTEREST

None declared.

## AUTHOR CONTRIBUTION

**Michelle McLellan:** Conceptualization (equal); Data curation (equal); Formal analysis (lead); Funding acquisition (supporting); Investigation (equal); Methodology (equal); Project administration (supporting); Resources (supporting); Writing‐original draft (lead); Writing‐review & editing (lead). **Bruce McLellan:** Conceptualization (equal); Data curation (equal); Funding acquisition (lead); Investigation (equal); Methodology (equal); Project administration (equal); Resources (equal); Writing‐review & editing (equal). **Rahel Sollmann:** Supervision (supporting); Writing‐review & editing (equal). **Heiko Wittmer:** Funding acquisition (supporting); Methodology (supporting); Supervision (lead); Writing‐review & editing (equal).

## Data Availability

Data are available at https://datadryad.org/stash/share/vuGxVQ‐HVBxBsg1bLnV4593oycNlmHHdS‐FCsuDVbt4
